# 5-Benzoyl-2-(1*H*-indol-3-yl)-4-(4-methyl­phen­yl)-4,5-dihydro­furan-3-carbonitrile

**DOI:** 10.1107/S1600536812011105

**Published:** 2012-03-21

**Authors:** J. Suresh, R. Vishnupriya, P. Gunasekaran, S. Perumal, P. L. Nilantha Lakshman

**Affiliations:** a Department of Physics, The Madura College, Madurai 625 011, India; b Department of Organic Chemistry, School of Chemistry, Madurai Kamaraj University, Madurai 625 021, India; c Department of Food Science and Technology, University of Ruhuna, Mapalana, Kamburupitiya 81100, Sri Lanka

## Abstract

The furan ring in the title compound, C_27_H_20_N_2_O_2_, adopts a twisted conformation about the *sp* ^3^—*sp* ^3^ bond. The mol­ecular structure is stabilized by an intra­molecular C—H⋯O inter­action which generates an *S*(6) ring motif. The crystal packing is stabilized by N—H⋯O and C—H⋯O inter­actions generating centrosymmetric *R*_2_^2^(18) and *C*(6) chain motifs, respectively. A weak C—H⋯π inter­action is also observed.

## Related literature

For the biological importance of furan derivatives, see: Auvin & Chabrier De Lassauniere (2005 ▶). For hydrogen-bonding graph-set notation, see: Bernstein *et al.* (1995 ▶). For additional conformation analysis, see: Cremer & Pople (1975 ▶).
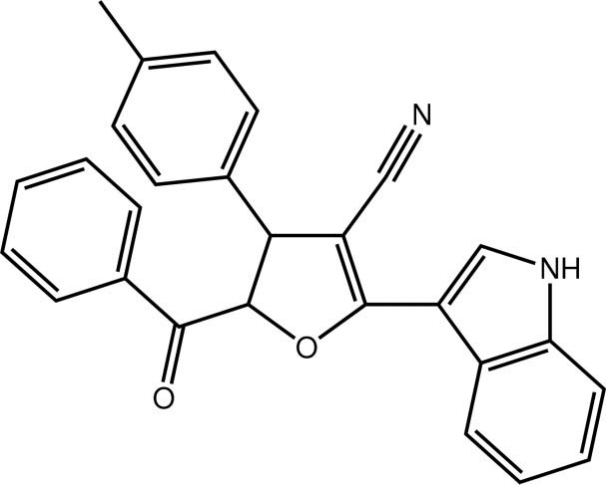


## Experimental

### 

#### Crystal data

C_27_H_20_N_2_O_2_
*M* *_r_* = 404.45
Monoclinic, 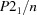

*a* = 9.8084 (4) Å
*b* = 15.9553 (7) Å
*c* = 13.8782 (7) Å
β = 107.185 (2)°
*V* = 2074.92 (16) Å^3^
*Z* = 4
Mo *K*α radiation
μ = 0.08 mm^−1^
*T* = 293 K
0.19 × 0.15 × 0.12 mm

#### Data collection

Bruker Kappa APEXII diffractometer
Absorption correction: multi-scan (*SADABS*; Sheldrick, 1996 ▶) *T*_min_ = 0.967, *T*_max_ = 0.974
21144 measured reflections
4647 independent reflections
3017 reflections with *I* > 2σ(*I*)
*R*_int_ = 0.038

#### Refinement

*R*[*F* ^2^ > 2σ(*F* ^2^)] = 0.045
*wR*(*F* ^2^) = 0.133
*S* = 1.02
4647 reflections
284 parameters
H atoms treated by a mixture of independent and constrained refinement
Δρ_max_ = 0.25 e Å^−3^
Δρ_min_ = −0.22 e Å^−3^

### 

Data collection: *APEX2* (Bruker, 2004 ▶); cell refinement: *SAINT* (Bruker, 2004 ▶); data reduction: *SAINT*; program(s) used to solve structure: *SHELXS97* (Sheldrick, 2008 ▶); program(s) used to refine structure: *SHELXL97* (Sheldrick, 2008 ▶); molecular graphics: *PLATON* (Spek, 2009 ▶); software used to prepare material for publication: *SHELXL97*.

## Supplementary Material

Crystal structure: contains datablock(s) global, I. DOI: 10.1107/S1600536812011105/tk5068sup1.cif

Structure factors: contains datablock(s) I. DOI: 10.1107/S1600536812011105/tk5068Isup2.hkl

Supplementary material file. DOI: 10.1107/S1600536812011105/tk5068Isup3.cml

Additional supplementary materials:  crystallographic information; 3D view; checkCIF report

## Figures and Tables

**Table 1:** Hydrogen-bond geometry (Å, °) *Cg*1 is the centroid of the C51–C56 ring.

*D*—H⋯*A*	*D*—H	H⋯*A*	*D*⋯*A*	*D*—H⋯*A*
C33—H33⋯O1	0.93	2.52	3.032 (2)	115
N2—H2⋯O2^i^	0.91 (2)	2.04 (2)	2.880 (2)	154
C44—H44⋯O2^ii^	0.93	2.55	3.329 (3)	142
C34—H34⋯*Cg*1^iii^	0.93	2.69	3.556 (3)	156

Symmetry codes: (i) 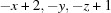; (ii) 
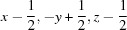
; (iii) 
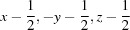
.

**Fig. 1.**
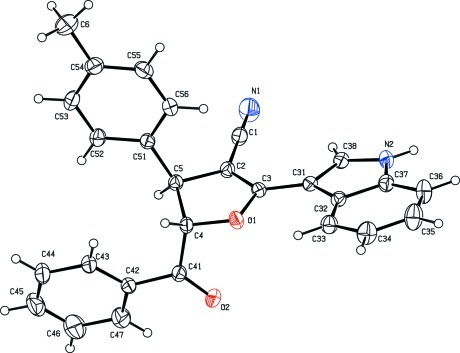
The molecular structure of (I), showing 40% probability displacement ellipsoids and the atom-numbering scheme.

**Fig. 2.**
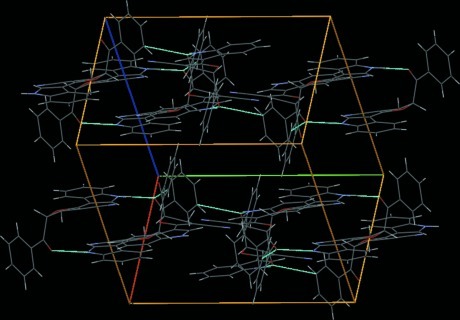
The partial packing diagram of (I). The C—H···O and N—H···O interactions are shown as blue lines.

## supplementary crystallographic information

### Comment

Furanyl derivatives have calplain-inhibiting activity and are used in the preparation of medicaments for the treatment of inflammatory and immunological diseases, cardiovascular and cerebro-vascular diseases, disorders of the central or peripheral nervous system, cachexia, osteoporosis, muscular dystrophy, proliferative diseases, cataracts, rejection reactions following organ transplants and auto-immune and viral diseases (Auvin *et al.*, 2005). The high medicinal value of these compounds in conjunction with our research interests prompted us to synthesize and report the X-ray structure of the title compound.

In the title compound (Fig 1), the five-membered furanyl ring adopts a twisted conformation as evident from the puckering parameters (Cremer & Pople, 1975) Q = 0.192 (2) Å and φ = 129.0 (6)°. The five-(N2/C38/C31/C32/C37) and six-membered (C32—C37) rings in the indole group are planar, with a dihedral angle of 0.74 (1)° between them. The dihedral angle between the phenyl rings (C42—C47 and C51—C56) is 15.24 (1)°.

Fig. 2 shows the partial packing of molecules in the crystal structure. The C—H···O and N—H···O intermolecular interactions generate C_1_^1^(6) chain and centrosymmetric *R*_2_^2^(18) motifs, respectively (Bernstein *et al.*, 1995). In addition, there is a weak C—H···π interaction, *viz*, C34—H34···*Cg*1^ii^_, _Table 1.

### Experimental

To a stirred mixture of 2-(1*H*-indole-3-carbonyl)-3-*p*-tolylacrylonitrile (1.0 molar eq.) and phenacylpyridinium bromide (1.0 molar eq.) in water (10 ml) was added drop wise triethylamine (0.25 molar eq.) at room temperature. The resulting clear solution, that slowly became turbid, was stirred at room temperature for 1 h. Then, the separated free flowing solid was filtered and washed with methanol (3 ml) to afford the title compound as a pale-yellow solid. The product thus obtained was recrystallized from an EtOH-EtOAc mixture (1:1 ratio *v*/*v* ml) to the give pure compound as pale-yellow crystals. Yield: 92%. *M*.pt: 502 K.

### Refinement

The H atoms were placed at calculated positions and allowed to ride on their carrier atoms with C—H = 0.93–0.98 Å, and with *U*_iso_ = 1.2–1.5*U*_eq_(C). The N-bound H atom was located in a difference Fourier map and refined freely.

### Crystal data

**Table:**

C_27_H_20_N_2_O_2_	*F*(000) = 848
*M**_r_* = 404.45	*D*_x_ = 1.295 Mg m^−^^3^
Monoclinic, *P*2_1_/*n*	Mo *K*α radiation, λ = 0.71073 Å
Hall symbol: -P 2yn	Cell parameters from 2000 reflections
*a* = 9.8084 (4) Å	θ = 2–31°
*b* = 15.9553 (7) Å	µ = 0.08 mm^−^^1^
*c* = 13.8782 (7) Å	*T* = 293 K
β = 107.185 (2)°	Block, pale-yellow
*V* = 2074.92 (16) Å^3^	0.19 × 0.15 × 0.12 mm
*Z* = 4	

### Data collection

**Table:**

Bruker Kappa APEXII diffractometer	4647 independent reflections
Radiation source: fine-focus sealed tube	3017 reflections with *I* > 2σ(*I*)
Graphite monochromator	*R*_int_ = 0.038
Detector resolution: 0 pixels mm^-1^	θ_max_ = 27.3°, θ_min_ = 2.0°
ω and φ scans	*h* = −12→12
Absorption correction: multi-scan (*SADABS*; Sheldrick, 1996)	*k* = −20→20
*T*_min_ = 0.967, *T*_max_ = 0.974	*l* = −17→17
21144 measured reflections	

### Refinement

**Table:**

Refinement on *F*^2^	Primary atom site location: structure-invariant direct methods
Least-squares matrix: full	Secondary atom site location: difference Fourier map
*R*[*F*^2^ > 2σ(*F*^2^)] = 0.045	Hydrogen site location: inferred from neighbouring sites
*wR*(*F*^2^) = 0.133	H atoms treated by a mixture of independent and constrained refinement
*S* = 1.02	*w* = 1/[σ^2^(*F*_o_^2^) + (0.0581*P*)^2^ + 0.5454*P*] where *P* = (*F*_o_^2^ + 2*F*_c_^2^)/3
4647 reflections	(Δ/σ)_max_ < 0.001
284 parameters	Δρ_max_ = 0.25 e Å^−^^3^
0 restraints	Δρ_min_ = −0.22 e Å^−^^3^

### Special details

**Table:**

Geometry. All e.s.d.'s (except the e.s.d. in the dihedral angle between two l.s. planes) are estimated using the full covariance matrix. The cell e.s.d.'s are taken into account individually in the estimation of e.s.d.'s in distances, angles and torsion angles; correlations between e.s.d.'s in cell parameters are only used when they are defined by crystal symmetry. An approximate (isotropic) treatment of cell e.s.d.'s is used for estimating e.s.d.'s involving l.s. planes.
Refinement. Refinement of *F*^2^ against ALL reflections. The weighted *R*-factor *wR* and goodness of fit *S* are based on *F*^2^, conventional *R*-factors *R* are based on *F*, with *F* set to zero for negative *F*^2^. The threshold expression of *F*^2^ > σ(*F*^2^) is used only for calculating *R*-factors(gt) *etc*. and is not relevant to the choice of reflections for refinement. *R*-factors based on *F*^2^ are statistically about twice as large as those based on *F*, and *R*- factors based on ALL data will be even larger.

### Fractional atomic coordinates and isotropic or equivalent isotropic displacement parameters (Å^2^)

**Table:**

	*x*	*y*	*z*	*U*_iso_*/*U*_eq_	
H2	1.231 (2)	−0.0946 (12)	0.5116 (14)	0.044 (5)*	
C1	0.7913 (2)	−0.08013 (11)	0.21842 (13)	0.0408 (4)	
C2	0.78765 (18)	0.00744 (10)	0.23169 (12)	0.0331 (4)	
C3	0.88408 (17)	0.05202 (10)	0.30178 (12)	0.0301 (4)	
C4	0.71584 (17)	0.14707 (10)	0.22283 (12)	0.0318 (4)	
H4	0.7178	0.1947	0.1787	0.038*	
C5	0.68263 (18)	0.06474 (10)	0.15994 (12)	0.0314 (4)	
H5	0.5848	0.0465	0.1531	0.038*	
C6	0.7683 (3)	0.09390 (17)	−0.23642 (17)	0.0734 (7)	
H6A	0.8681	0.0883	−0.2296	0.110*	
H6B	0.7350	0.1474	−0.2658	0.110*	
H6C	0.7163	0.0501	−0.2791	0.110*	
C31	1.01403 (17)	0.02910 (10)	0.37695 (12)	0.0310 (4)	
C32	1.12543 (17)	0.08399 (11)	0.43391 (12)	0.0325 (4)	
C33	1.14643 (19)	0.17039 (11)	0.44071 (14)	0.0405 (4)	
H33	1.0781	0.2066	0.4014	0.049*	
C34	1.2696 (2)	0.20113 (13)	0.50646 (16)	0.0535 (5)	
H34	1.2841	0.2588	0.5116	0.064*	
C35	1.3735 (2)	0.14808 (14)	0.56565 (17)	0.0615 (6)	
H35	1.4558	0.1709	0.6097	0.074*	
C36	1.3567 (2)	0.06253 (14)	0.56019 (15)	0.0527 (5)	
H36	1.4263	0.0268	0.5991	0.063*	
C37	1.23189 (18)	0.03164 (11)	0.49441 (13)	0.0367 (4)	
C38	1.05756 (18)	−0.05100 (11)	0.40606 (13)	0.0357 (4)	
H38	1.0052	−0.0992	0.3819	0.043*	
C41	0.60327 (18)	0.16108 (10)	0.27659 (13)	0.0337 (4)	
C42	0.45710 (18)	0.18082 (11)	0.21168 (13)	0.0372 (4)	
C43	0.4312 (2)	0.21107 (12)	0.11489 (14)	0.0463 (5)	
H43	0.5073	0.2244	0.0906	0.056*	
C44	0.2925 (2)	0.22178 (14)	0.05363 (17)	0.0619 (6)	
H44	0.2753	0.2419	−0.0118	0.074*	
C45	0.1806 (3)	0.20254 (16)	0.0902 (2)	0.0759 (8)	
H45	0.0873	0.2089	0.0490	0.091*	
C46	0.2051 (3)	0.17414 (18)	0.1867 (2)	0.0786 (8)	
H46	0.1287	0.1622	0.2112	0.094*	
C47	0.3427 (2)	0.16313 (14)	0.24769 (17)	0.0574 (6)	
H47	0.3589	0.1437	0.3133	0.069*	
C51	0.70509 (18)	0.07130 (10)	0.05677 (12)	0.0320 (4)	
C52	0.59093 (19)	0.08565 (11)	−0.02828 (13)	0.0380 (4)	
H52	0.4993	0.0900	−0.0222	0.046*	
C53	0.6116 (2)	0.09362 (12)	−0.12209 (14)	0.0447 (5)	
H53	0.5334	0.1032	−0.1782	0.054*	
C54	0.7455 (2)	0.08765 (12)	−0.13439 (15)	0.0465 (5)	
C55	0.8592 (2)	0.07429 (13)	−0.04929 (16)	0.0503 (5)	
H55	0.9509	0.0707	−0.0555	0.060*	
C56	0.8396 (2)	0.06610 (12)	0.04483 (14)	0.0426 (4)	
H56	0.9181	0.0570	0.1009	0.051*	
N1	0.7928 (2)	−0.15113 (11)	0.20826 (15)	0.0670 (6)	
N2	1.18746 (16)	−0.04969 (10)	0.47495 (11)	0.0387 (4)	
O1	0.85398 (12)	0.13585 (7)	0.29520 (9)	0.0369 (3)	
O2	0.62895 (14)	0.14959 (8)	0.36623 (9)	0.0452 (3)	

### Atomic displacement parameters (Å^2^)

**Table:**

	*U*^11^	*U*^22^	*U*^33^	*U*^12^	*U*^13^	*U*^23^
C1	0.0479 (11)	0.0332 (10)	0.0347 (10)	0.0026 (8)	0.0019 (8)	−0.0008 (8)
C2	0.0382 (9)	0.0284 (8)	0.0297 (9)	0.0029 (7)	0.0054 (7)	0.0004 (7)
C3	0.0333 (9)	0.0274 (8)	0.0293 (9)	0.0050 (7)	0.0086 (7)	0.0024 (7)
C4	0.0329 (9)	0.0289 (9)	0.0287 (9)	0.0038 (6)	0.0014 (7)	0.0007 (7)
C5	0.0322 (9)	0.0284 (8)	0.0306 (9)	0.0002 (6)	0.0047 (7)	−0.0014 (7)
C6	0.0889 (19)	0.0920 (19)	0.0465 (14)	0.0128 (14)	0.0310 (13)	0.0068 (12)
C31	0.0336 (9)	0.0323 (9)	0.0273 (9)	0.0033 (7)	0.0093 (7)	0.0010 (7)
C32	0.0326 (9)	0.0384 (9)	0.0272 (9)	0.0009 (7)	0.0099 (7)	0.0021 (7)
C33	0.0412 (10)	0.0380 (10)	0.0403 (11)	−0.0008 (8)	0.0091 (8)	0.0031 (8)
C34	0.0522 (13)	0.0450 (12)	0.0566 (13)	−0.0126 (9)	0.0057 (10)	−0.0017 (10)
C35	0.0471 (13)	0.0626 (15)	0.0604 (15)	−0.0160 (10)	−0.0061 (11)	0.0037 (11)
C36	0.0389 (11)	0.0599 (13)	0.0495 (12)	−0.0012 (9)	−0.0021 (9)	0.0106 (10)
C37	0.0345 (10)	0.0417 (10)	0.0328 (9)	0.0018 (7)	0.0083 (8)	0.0054 (7)
C38	0.0385 (10)	0.0363 (9)	0.0306 (9)	0.0027 (7)	0.0075 (8)	0.0002 (7)
C41	0.0402 (10)	0.0257 (8)	0.0306 (9)	0.0035 (7)	0.0031 (7)	−0.0009 (7)
C42	0.0365 (10)	0.0361 (9)	0.0343 (10)	0.0075 (7)	0.0030 (8)	−0.0048 (7)
C43	0.0479 (11)	0.0472 (11)	0.0369 (11)	0.0155 (9)	0.0020 (9)	−0.0002 (8)
C44	0.0636 (15)	0.0593 (14)	0.0459 (13)	0.0249 (11)	−0.0098 (11)	−0.0050 (10)
C45	0.0428 (13)	0.0837 (18)	0.081 (2)	0.0188 (12)	−0.0127 (13)	−0.0223 (15)
C46	0.0410 (13)	0.105 (2)	0.085 (2)	0.0046 (13)	0.0114 (13)	−0.0088 (16)
C47	0.0429 (12)	0.0740 (15)	0.0535 (13)	0.0057 (10)	0.0115 (10)	0.0012 (11)
C51	0.0358 (9)	0.0263 (8)	0.0310 (9)	0.0028 (7)	0.0052 (7)	−0.0013 (7)
C52	0.0342 (10)	0.0407 (10)	0.0360 (10)	−0.0023 (7)	0.0054 (8)	−0.0015 (8)
C53	0.0501 (12)	0.0482 (11)	0.0300 (10)	−0.0001 (9)	0.0029 (9)	0.0024 (8)
C54	0.0597 (13)	0.0430 (11)	0.0390 (11)	0.0073 (9)	0.0180 (10)	0.0016 (8)
C55	0.0473 (12)	0.0582 (13)	0.0499 (12)	0.0167 (9)	0.0210 (10)	0.0063 (10)
C56	0.0373 (10)	0.0482 (11)	0.0391 (11)	0.0107 (8)	0.0062 (8)	0.0030 (8)
N1	0.0830 (14)	0.0337 (10)	0.0688 (13)	0.0048 (9)	−0.0014 (11)	−0.0071 (8)
N2	0.0395 (8)	0.0369 (8)	0.0357 (8)	0.0085 (7)	0.0051 (7)	0.0084 (7)
O1	0.0331 (6)	0.0279 (6)	0.0415 (7)	0.0036 (5)	−0.0016 (5)	−0.0029 (5)
O2	0.0515 (8)	0.0508 (8)	0.0290 (7)	0.0095 (6)	0.0051 (6)	0.0042 (6)

### Geometric parameters (Å, º)

**Table:**

C1—N1	1.142 (2)	C37—N2	1.370 (2)
C1—C2	1.411 (2)	C38—N2	1.348 (2)
C2—C3	1.343 (2)	C38—H38	0.9300
C2—C5	1.511 (2)	C41—O2	1.208 (2)
C3—O1	1.3670 (18)	C41—C42	1.484 (2)
C3—C31	1.436 (2)	C42—C43	1.379 (3)
C4—O1	1.4397 (19)	C42—C47	1.386 (3)
C4—C41	1.521 (2)	C43—C44	1.385 (3)
C4—C5	1.557 (2)	C43—H43	0.9300
C4—H4	0.9800	C44—C45	1.374 (3)
C5—C51	1.515 (2)	C44—H44	0.9300
C5—H5	0.9800	C45—C46	1.366 (4)
C6—C54	1.501 (3)	C45—H45	0.9300
C6—H6A	0.9600	C46—C47	1.376 (3)
C6—H6B	0.9600	C46—H46	0.9300
C6—H6C	0.9600	C47—H47	0.9300
C31—C38	1.370 (2)	C51—C56	1.380 (2)
C31—C32	1.441 (2)	C51—C52	1.385 (2)
C32—C33	1.393 (2)	C52—C53	1.381 (3)
C32—C37	1.405 (2)	C52—H52	0.9300
C33—C34	1.372 (3)	C53—C54	1.377 (3)
C33—H33	0.9300	C53—H53	0.9300
C34—C35	1.391 (3)	C54—C55	1.381 (3)
C34—H34	0.9300	C55—C56	1.381 (3)
C35—C36	1.374 (3)	C55—H55	0.9300
C35—H35	0.9300	C56—H56	0.9300
C36—C37	1.383 (3)	N2—H2	0.91 (2)
C36—H36	0.9300		
			
N1—C1—C2	179.0 (2)	N2—C38—C31	109.98 (15)
C3—C2—C1	125.41 (15)	N2—C38—H38	125.0
C3—C2—C5	110.74 (14)	C31—C38—H38	125.0
C1—C2—C5	123.49 (15)	O2—C41—C42	121.82 (17)
C2—C3—O1	112.21 (14)	O2—C41—C4	121.48 (15)
C2—C3—C31	132.51 (15)	C42—C41—C4	116.40 (14)
O1—C3—C31	115.18 (14)	C43—C42—C47	119.15 (18)
O1—C4—C41	110.25 (13)	C43—C42—C41	122.17 (17)
O1—C4—C5	106.50 (12)	C47—C42—C41	118.55 (17)
C41—C4—C5	109.70 (13)	C42—C43—C44	120.4 (2)
O1—C4—H4	110.1	C42—C43—H43	119.8
C41—C4—H4	110.1	C44—C43—H43	119.8
C5—C4—H4	110.1	C45—C44—C43	119.5 (2)
C2—C5—C51	113.80 (13)	C45—C44—H44	120.2
C2—C5—C4	98.75 (12)	C43—C44—H44	120.2
C51—C5—C4	113.99 (13)	C46—C45—C44	120.5 (2)
C2—C5—H5	109.9	C46—C45—H45	119.7
C51—C5—H5	109.9	C44—C45—H45	119.7
C4—C5—H5	109.9	C45—C46—C47	120.2 (2)
C54—C6—H6A	109.5	C45—C46—H46	119.9
C54—C6—H6B	109.5	C47—C46—H46	119.9
H6A—C6—H6B	109.5	C46—C47—C42	120.2 (2)
C54—C6—H6C	109.5	C46—C47—H47	119.9
H6A—C6—H6C	109.5	C42—C47—H47	119.9
H6B—C6—H6C	109.5	C56—C51—C52	117.94 (16)
C38—C31—C3	125.75 (15)	C56—C51—C5	121.36 (15)
C38—C31—C32	106.58 (15)	C52—C51—C5	120.67 (15)
C3—C31—C32	127.64 (15)	C53—C52—C51	120.77 (17)
C33—C32—C37	118.61 (16)	C53—C52—H52	119.6
C33—C32—C31	135.37 (16)	C51—C52—H52	119.6
C37—C32—C31	106.01 (15)	C54—C53—C52	121.43 (18)
C34—C33—C32	118.84 (17)	C54—C53—H53	119.3
C34—C33—H33	120.6	C52—C53—H53	119.3
C32—C33—H33	120.6	C53—C54—C55	117.63 (18)
C33—C34—C35	121.53 (19)	C53—C54—C6	121.72 (19)
C33—C34—H34	119.2	C55—C54—C6	120.64 (19)
C35—C34—H34	119.2	C54—C55—C56	121.37 (19)
C36—C35—C34	121.10 (19)	C54—C55—H55	119.3
C36—C35—H35	119.4	C56—C55—H55	119.3
C34—C35—H35	119.4	C51—C56—C55	120.85 (17)
C35—C36—C37	117.31 (18)	C51—C56—H56	119.6
C35—C36—H36	121.3	C55—C56—H56	119.6
C37—C36—H36	121.3	C38—N2—C37	109.46 (14)
N2—C37—C36	129.44 (17)	C38—N2—H2	125.2 (12)
N2—C37—C32	107.95 (15)	C37—N2—H2	124.4 (12)
C36—C37—C32	122.60 (17)	C3—O1—C4	107.96 (12)
			
N1—C1—C2—C3	106 (14)	C5—C4—C41—O2	105.78 (18)
N1—C1—C2—C5	−82 (14)	O1—C4—C41—C42	175.00 (13)
C1—C2—C3—O1	178.88 (16)	C5—C4—C41—C42	−68.02 (17)
C5—C2—C3—O1	5.6 (2)	O2—C41—C42—C43	166.10 (17)
C1—C2—C3—C31	2.8 (3)	C4—C41—C42—C43	−20.1 (2)
C5—C2—C3—C31	−170.48 (17)	O2—C41—C42—C47	−18.1 (3)
C3—C2—C5—C51	106.09 (16)	C4—C41—C42—C47	155.73 (17)
C1—C2—C5—C51	−67.3 (2)	C47—C42—C43—C44	−1.5 (3)
C3—C2—C5—C4	−15.07 (18)	C41—C42—C43—C44	174.33 (17)
C1—C2—C5—C4	171.50 (16)	C42—C43—C44—C45	0.4 (3)
O1—C4—C5—C2	18.97 (16)	C43—C44—C45—C46	0.9 (4)
C41—C4—C5—C2	−100.33 (14)	C44—C45—C46—C47	−1.1 (4)
O1—C4—C5—C51	−102.04 (15)	C45—C46—C47—C42	0.0 (4)
C41—C4—C5—C51	138.65 (14)	C43—C42—C47—C46	1.3 (3)
C2—C3—C31—C38	−12.6 (3)	C41—C42—C47—C46	−174.7 (2)
O1—C3—C31—C38	171.41 (15)	C2—C5—C51—C56	−32.8 (2)
C2—C3—C31—C32	165.29 (18)	C4—C5—C51—C56	79.46 (19)
O1—C3—C31—C32	−10.7 (2)	C2—C5—C51—C52	149.49 (15)
C38—C31—C32—C33	−179.13 (19)	C4—C5—C51—C52	−98.29 (18)
C3—C31—C32—C33	2.7 (3)	C56—C51—C52—C53	0.6 (3)
C38—C31—C32—C37	0.53 (18)	C5—C51—C52—C53	178.47 (16)
C3—C31—C32—C37	−177.68 (16)	C51—C52—C53—C54	0.0 (3)
C37—C32—C33—C34	−0.3 (3)	C52—C53—C54—C55	−0.7 (3)
C31—C32—C33—C34	179.29 (19)	C52—C53—C54—C6	178.30 (19)
C32—C33—C34—C35	0.3 (3)	C53—C54—C55—C56	0.8 (3)
C33—C34—C35—C36	0.2 (4)	C6—C54—C55—C56	−178.2 (2)
C34—C35—C36—C37	−0.7 (3)	C52—C51—C56—C55	−0.5 (3)
C35—C36—C37—N2	−179.33 (19)	C5—C51—C56—C55	−178.35 (16)
C35—C36—C37—C32	0.6 (3)	C54—C55—C56—C51	−0.2 (3)
C33—C32—C37—N2	179.83 (15)	C31—C38—N2—C37	1.1 (2)
C31—C32—C37—N2	0.10 (19)	C36—C37—N2—C38	179.26 (19)
C33—C32—C37—C36	−0.1 (3)	C32—C37—N2—C38	−0.7 (2)
C31—C32—C37—C36	−179.88 (17)	C2—C3—O1—C4	7.86 (18)
C3—C31—C38—N2	177.26 (15)	C31—C3—O1—C4	−175.33 (14)
C32—C31—C38—N2	−0.99 (19)	C41—C4—O1—C3	101.52 (14)
O1—C4—C41—O2	−11.2 (2)	C5—C4—O1—C3	−17.43 (17)

### Hydrogen-bond geometry (Å, º)

Cg1 is the centroid of the C51–C56 ring.

**Table:**

*D*—H···*A*	*D*—H	H···*A*	*D*···*A*	*D*—H···*A*
C33—H33···O1	0.93	2.52	3.032 (2)	115
N2—H2···O2^i^	0.91 (2)	2.04 (2)	2.880 (2)	154
C44—H44···O2^ii^	0.93	2.55	3.329 (3)	142
C34—H34···*Cg*1^iii^	0.93	2.69	3.556 (3)	156

Symmetry codes: (i) −*x*+2, −*y*, −*z*+1; (ii) *x*−1/2, −*y*+1/2, *z*−1/2; (iii) *x*−1/2, −*y*−1/2, *z*−1/2.
